# The Association Between Korean American Nurse and Primary Care Provider Burnout, Areas of Worklife, and Perceptions of Pandemic Experience: Cross-sectional Study

**DOI:** 10.2196/42490

**Published:** 2023-03-08

**Authors:** Linda Y Kim, Adrienne Martinez-Hollingworth, Harriet Aronow, Isa Caffe, Wenrui Xu, Christine Khanbijian, Mason Lee, Bernice Coleman, Angela Jun

**Affiliations:** 1 Nursing Research Cedars Sinai Medical Center Los Angeles, CA United States; 2 College of Nursing Samuel Merritt University Oakland, CA United States; 3 Sue and Bill School of Nursing University of California, Irvine Irvine, CA United States

**Keywords:** Korean American, registered nurses, primary care providers, burnout, pandemic, work environment

## Abstract

**Background:**

Korean immigrants are among the fastest-growing ethnic minority groups and make up the fifth-largest Asian group in the United States. A better understanding of the work environment factors and its impact on Korean American nurse and primary care provider (PCP) burnout may guide the development of targeted strategies to help mitigate burnout and workplace stressors, which is critical for the retention of Korean American nurses and PCPs to promote better alignment of national demographic trends and meet patients’ preference for cultural congruence with their health care providers (HCPs). Although there is a growing number of studies on HCP burnout, a limited number of studies specifically focus on the experience of ethnic minority HCPs, particularly during the COVID-19 pandemic.

**Objective:**

In light of these gaps in literature, the aim of this study was to assess burnout among Korean American HCPs and to identify work conditions during a pandemic that may be associated with Korean American nurse and PCP burnout.

**Methods:**

A total of 184 Korean American HCPs (registered nurses [RNs]: n=97; PCPs: n=87) practicing in Southern California responded to a web-based survey between February and April 2021. The Maslach Burnout Inventory, Areas of Worklife Survey, and Pandemic Experience & Perceptions Survey were used to measure burnout and work environment factors during the pandemic. A multivariate linear regression analysis was used to assess work environment factors associated with the 3 subcategories of burnout.

**Results:**

No significant differences were found in the level of burnout experienced by Korean American nurses and PCPs. For RNs, greater workload (*P*<.001), lower resource availability (*P*=.04), and higher risk perception (*P*=.02) were associated with higher emotional exhaustion. Greater workload was also associated with higher depersonalization (*P*=.003), whereas a greater (professional) community (*P*=.03) and higher risk perception (*P*=.006) were associated with higher personal accomplishment. For PCPs, greater workload and poor work-life balance were associated with higher emotional exhaustion (workload: *P*<.001; worklife: *P*=.005) and depersonalization (workload: *P*=.01; worklife: *P*<.001), whereas only reward was associated with personal accomplishment (*P*=.006).

**Conclusions:**

Findings from this study underscore the importance of strategies to promote a healthy work environment across multiple levels that recognize demographic variation among Korean American RNs and PCPs, potentially influencing their burnout mitigation needs. A growing recognition of identity-informed burnout experiences across frontline Korean American RNs and PCPs argues for future explorations that capture nuance both across and within this and other ethnic minority nurse and PCP groups. By recognizing and capturing these variations, we may better support the creation of targeted, burnout-mitigating strategies for all.

## Introduction

### Background

Even before the COVID-19 pandemic, 35% to 45% of nurses and 40% to 54% of physicians in the United States reported experiencing burnout [1-5]. Higher levels of burnout have been linked to lower job satisfaction and higher job turnover, as well as increased medical errors, poor patient health outcomes, and lower patient satisfaction scores [3,6-8]. Burnout may also threaten clinicians’ own health over the long term [9,10].

### Updating the Quadruple Aim

The triple aim, which sought to enhance patient experience and improve population health while reducing costs, was expanded to the quadruple aim, recognizing the critical threat of burnout among health care providers (HCPs) [11]. Most recently, a proposed “quintuple aim” suggests that the future of health care involves the early recognition and mitigation of health disparities [12]. This push, as well as emergent work on differential experiences of burnout among providers of color [13], argues for an expanded and nuanced view of HCP burnout that is contextualized within their cultural, racial/ethnic, and other socially informed or self-selected group memberships, such as the Korean American HCP group who often gets lumped with other Asian American groups [14], despite being one of the largest and fastest-growing Asian American groups.

### A Growing Need for Korean American Providers

In 2020, approximately 24 million US residents self-identified as Asian. Of these 24 million US residents, approximately 1.9 million self-identified as Korean [15]. Korean immigrants are among the fastest-growing ethnic minority groups and make up the fifth-largest Asian group in the United States [16].

Studies across the health care literature have demonstrated that ethnic minority adults, including Korean American individuals, display a preference for providers from their background, those able to speak their native language, and who are familiar with historical challenges to accessing needed service [17]. Given this preference for cultural congruence, the need for Korean American HCPs has never been greater and is growing in alignment with demographic trends nationally [17]. Critical to the retention of Korean American HCPs is an understanding of what factors burn them out, potentially impacting their longevity in the field.

### Impact of Discrimination on Korean American Provider Well-being During COVID-19

Discrimination, bullying, and incivility, especially in the workplace, undermine the culture of safety for both HCPs and patients alike. As such, in 2018, the Joint Commission has issued a Sentinel Event Alert on the physical and verbal violence against health care workers [18]. Numerous studies have found that mistreatment and discrimination toward HCPs are associated with higher levels of HCP burnout [19,20]. Additionally, a study on North American Asian HCPs and their experiences of discrimination during the COVID-19 pandemic demonstrated a surge of microaggressions related to the pandemic and a lack of institutional and public acknowledgment of the issue [21].

### Aspects of the Work Environment Impacting Burnout

The Job Demands-Resources Theory suggests that job strain occurs when there is a mismatch between the demands placed on an individual and their ability to meet those demands [22-24]. It considers a wide range of both positive and negative work environment factors with indicators of employee well-being. Hence, burnout is often the result of high job demands including heavy workload, role ambiguity, role conflict, role stress, stressful events, and work pressure, among others [22-24]. As such, work environment factors such as work process inefficiencies; excessive workloads; organizational climate factors; and deterioration in control, autonomy, and meaning at work have been associated with burnout among physicians and nurses [1,5,25-28].

### Aim of the Study

Although there is a growing number of studies on HCP burnout, a limited number of studies specifically focus on the experience of ethnic minority HCPs, particularly during the COVID-19 pandemic. A better understanding of the work environment factors that cause a mismatch between the demands placed on the HCPs and their ability to meet those demands leading to burnout may guide the development of targeted strategies to help mitigate HCP burnout. In light of these gaps in literature, the aim of this study was to assess burnout among Korean American HCPs and to identify work conditions during a pandemic that may be associated with Korean American nurse and primary care provider (PCP) burnout.

## Methods

### Study Population and Recruitment

Registered nurses (RNs), nurse practitioners (NPs), Doctors of Medicine (MDs), and physician assistants (PAs) who provide direct patient care in Southern California were eligible to participate. The participants were grouped into RNs or PCPs (NPs, MDs, and PAs) based on their scope of practice. The decision to organize providers into these groups was informed by previous studies, which have shown that burnout levels are impacted by specific patient care tasks performed by different members of the health care team [29,30]. Although NPs are also RNs, they were grouped with PCPs since their scope of practice more closely aligns with PCPs, including but not limited to diagnosing patient conditions, initiating or managing medical treatment, and prescribing medications or nonpharmacologic treatments [31].

Recruitment methods included the distribution of an informational flyer (in English) via email to approximately 196 RN and NP members of the Korean American Nurses Association of Southern California and approximately 4440 Korean American PCPs (MDs and PAs) within the Seoul Medical Group, Independent Practitioner Association; 200 members of the Korean American Medical Association of Southern California; and 40 members of the Korean American Graduate Medical Association. Recruitment fliers (in English) were also used as an advertisement through the social media platforms Facebook, Instagram, and KakaoTalk. Additional participants were referred to the study via word of mouth from participants of the study.

### Measures

#### Demographic and Employment Information

Demographic information included participant gender, age (in years), education level, professional degree, tenure (years of work experience), work setting (acute care hospital or medical center vs ambulatory or outpatient care setting vs skilled nursing facility vs both acute care and another setting vs other), and marital status.

#### Burnout

The Maslach Burnout Inventory–Human Services Survey (MBI-HSS) was found to be valid and reliable in previous studies evaluating employee burnout [32]. The MBI-HSS includes 22 items and 3 subscales (emotional exhaustion=9 items, depersonalization=5 items, and personal accomplishment=8 items). Response options for each of the 9 items ranged from “never” (0) to “everyday” (6) with a total possible average score ranging from 0-6.

#### Areas of Worklife

The Areas of Worklife Survey (AWS) is a survey created to assess employees’ perceptions of work setting qualities that play a role in whether they experience work engagement or burnout. These work setting qualities include workload (amount of work), control (opportunity to make choices and decisions, solve problems, and contribute to the fulfillment of responsibilities), reward (recognition—financial and social—for contributions on the job), community (organization’s social environment or communities characterized by support, collaboration, and positive feelings), fairness (extent to which the organization has consistent and equitable rules for everyone), and values (what is important to the organization and to its members) [33]. A mismatch between an individual and their perceptions of the work setting may lead to emotional exhaustion, cynicism, and inefficacy of burnout, whereas a match would indicate higher engagement with one’s work [33]. The AWS has been used in conjunction with the MBI to assess the work environment factors associated with burnout and has demonstrated reliability and validity across a variety of occupational settings [33]. The AWS includes 28 items and 6 subscales (workload=5 items, control=4 items, reward=4 items, community=5 items, fairness=6 items, and values=4 items), measured on a 5‐point Likert scale, rated from 1 (strongly disagree) to 5 (strongly agree).

#### Pandemic Experience and Perceptions

The Pandemic Experience & Perceptions Survey (PEPS) assesses the perception of work settings specifically during pandemics, such as COVID-19, that impacts the way people work, with potential implications for employees’ health, well-being, and work engagement [34]. The survey includes a total of 35 items and 6 subscales (impact=3 items, resources=5 items, risk perception=7 items, worklife=7 items, leadership=12 items, and work setting=1 item). For the purposes of this study, only 23 items from the PEPS were included: items 4-8 (resources: rated on a 1-5 scale ranging from “completely inadequate” to “completely adequate”), items 9-14 (risk perception: rated on a 1-5 scale ranging from “no risk at all” to “life-threatening risk”), items 16-22 (worklife: rated on a 1-5 scale ranging from “strongly disagree” to “strongly agree”), and items 23-27 (leadership: rated on a 1-5 scale ranging from “not at all” to “frequently, if not always”).

### Data Collection

Data were collected on the web between February and April 2021, using the REDCap (Research Electronic Data Capture; Vanderbilt University) system. Participants completed the electronic survey, which includes items from the demographic questionnaire, MBI-HSS, AWS, and PEPS. Each participant was assigned a unique record ID number automatically by REDCap upon the completion of the survey. The only identifiable data record linking the participant to the record ID number was their email address. The principal investigator reviewed all data collection forms on an ongoing basis for data completeness, duplication, and accuracy as well as protocol compliance.

### Statistical Analysis

Univariate analysis included descriptive statistics used to analyze participant demographics, levels of burnout, areas of worklife, and pandemic experience and perception. To assess for differences between RNs and PCPs, 2-tailed *t* tests were used for continuous variables and chi-square analyses were used for categorical variables. Bivariate Pearson correlation analysis was used to explore statistically significant factors associated with the 3 subcategories of burnout. All results with *P*<.05 were included in the multivariate linear regression analysis using the backward elimination method, to develop the most parsimonious final model to assess for factors associated with the 3 subcategories of burnout. All analysis was conducted using SPSS statistical software (version 24; IBM Corp).

### Ethics Approval

This study was performed in line with the principles of the Declaration of Helsinki. Approval was granted by the Ethics Committee of Cedars Sinai Medical Center (STUDY00000931; approved on August 27, 2020). Informed consent was obtained from all individual participants included in the study.

## Results

### Univariate Analysis Results: Participant Demographics, Burnout, Areas of Worklife, and Pandemic Experience and Perceptions

Table 1 describes responses from 184 Korean American HCPs (RNs: n=97; PCPs: n=87). Most of the participants were female (RNs: 76/97, 78%; PCPs: 72/87, 83%) and married (RNs: 59/97, 61%, PCPs: 58/87, 67%). The average age was 38.9 (SD 9.9) years for RNs and 41.5 (SD 9.8) years for PCPs. A majority of RNs (63/97, 65%) reported working in acute care settings with average of 4.6 years of work experience, whereas a majority of PCPs (62/87, 71%) reported working in ambulatory or outpatient care settings with an average of 5.3 years of work experience. Although no statistically significant differences were seen in emotional exhaustion (*P*=.85) or depersonalization (*P*=.52) between the 2 HCP groups, PCPs reported statistically significant higher levels of personal accomplishment (*P*=.03). There were also no significant differences in their perceptions of workload (*P*=.99), control (*P*=.33), community (*P*=.84), fairness (*P*=.30), value (*P*=.86), worklife (*P*=.10), and leadership (*P*=.08); however, significant differences were noted in reward (*P*<.001), resource availability (*P*=.03), and risk perception (*P*=.02).

**Table 1:** Univariate analysis: participant demographics, burnout, areas of worklife, and pandemic experience and perceptions.

Variable			RN^a^ (n=97)		PCP^b^ (n=87)		*P* value	
**Gender, n (%)**								.45
	Female	76 (78)		72 (83)				
	Male	21 (22)		15 (17)				
Age (years), mean (SD)			38.9 (9.9)		41.5 (9.8)		.08	
**Education, n (%)**								<.001
	Associate’s degree	12 (12)		0 (0)				
	Bachelor’s degree	68 (70)		2 (2)				
	Master’s degree	17 (18)		66 (76)				
	PhD^c^, MD^d^, or other doctoral degree	0 (0)		19 (22)				
**Marital status, n (%)^e^**								.69
	Divorced	1 (1)		0 (0)				
	Married	59 (61)		58 (67)				
	Single	36 (37)		28 (32)				
	Widowed	1 (1)		1 (1)				
Work experience or tenure (years), mean (SD)			4.6 (3.9)		5.3 (6.5)		.68	
**Work setting, n (%)^e^**								<.001
	Acute care hospital or medical center	63 (65)		19 (22)				
	Ambulatory or outpatient care setting	20 (21)		62 (71)				
	SNF^f^	8 (8)		0 (0)				
	Both (acute and ambulatory or SNF)	0 (0)		6 (7)				
	Other (eg, public health or academic)	6 (6)		0 (0)				
**Burnout (0-6 scale), mean (SD)**								
	Emotional exhaustion	3.2 (1.4)		3.3 (1.4)		.85		
	Depersonalization	2.2 (1.3)		2.3 (1.4)		.52		
	Personal accomplishment	3.9 (1.0)		4.2 (1.0)		.03		
**Areas of worklife** **(1-5 scale)** **, mean (SD)**								
	Workload	3.2 (0.7)		3.2 (0.7)		.99		
	Control	3.6 (0.7)		3.7 (0.9)		.33		
	Reward	2.6 (0.6)		2.9 (0.6)		<.001		
	Community	3.6 (0.6)		3.6 (0.7)		.84		
	Fairness	3.0 (0.7)		3.1 (0.7)		.30		
	Value	3.5 (0.7)		3.5 (0.8)		.86		
**Pandemic experience and perceptions** **(1-5 scale)** **, mean (SD)**								
	Resource availability	3.6 (0.9)		3.9 (0.8)		.03		
	Risk perception	2.6 (0.5)		2.4 (0.5)		.02		
	Worklife	3.4 (0.7)		3.6 (0.7)		.10		
	Leadership	3.3 (0.9)		3.5 (0.8)		.08		

^a^RN: registered nurse.

^b^PCP: primary care provider.

^c^PhD: Doctor of Philosophy.

^d^MD: Doctor of Medicine.

^e^Data may not add up to 100% due to rounding.

^f^SNF: skilled nursing facility.

### Bivariate Pearson Correlation Analysis Results (RN Versus PCP): Association Between Burnout, Areas of Worklife, and Pandemic Experiences and Perceptions

Multimedia Appendix 1 describes the statistically significant factors associated with the 3 subcategories of burnout. The variables found to be statistically significant (*P*<.05) were entered into the multivariate linear regression models.

### Multivariate Linear Regression Analysis Results: Association Between Areas of Worklife, Pandemic Experience and Perceptions, and Burnout

Table 2 describes the statistically significant areas of worklife and pandemic experience and perceptions factors associated with the 3 subcategories of burnout. For RNs, greater workload (*P*<.001), lower resource availability (*P*=.04), and higher risk perception (*P*=.02) were associated with higher emotional exhaustion. Greater workload was also associated with higher depersonalization (*P*=.003), whereas a greater (professional) community (*P*=.03) and higher risk perception (*P*=.006) were associated with higher personal accomplishment. For PCPs, greater workload (*P*<.001) and poor work-life balance (*P*=.005) were associated with higher emotional exhaustion (workload: *P*<.001; worklife: *P*=.005) and depersonalization (workload: *P*=.01; worklife: *P*<.001), whereas only reward was associated with personal accomplishment (*P*=.006).

**Table 2:** Multivariate linear regression analysis: association between areas of worklife, pandemic experience and perceptions, and burnout^a^.

Burnout subcategory, associated factor			RN^b^ (n=97)							PCP^c^ (n=87)				
			Estimate (SE)		95% CI		*P* value		Estimate (SE)			95% CI		*P* value
**Emotional exhaustion (RN: adjusted *R^2^*=.321; PCP: adjusted *R^2^*=.303)**														
	Workload	.344 (.189)		.319 to .065		<.001		.424 (.183)			.453 to 1.179		<.001	
	Reward	–.195 (.225)		–.882 to .012		.06		—^d^			—		—	
	Resource availability	–.236 (.176)		–.714 to –.016		.04		—			—		—	
	Risk perception	.218 (.219)		.102 to .973		.02		—			—		—	
	Worklife	.217 (.234)		–.030 to –.898		.07		–.273 (.189)			–.920 to –.169		.005	
**Depersonalization (RN: adjusted *R^2^*=.069; PCP: adjusted *R^2^*=.311)**														
	Workload	.302 (.187)		.207 to .948		.003		.208 (.197)			.013 to .798		.01	
	Worklife	—		—		—		–.379 (.204)			–1.172 to –.360		<.000	
**Personal accomplishment (RN: adjusted *R^2^*=.134; PCP: adjusted *R^2^*=.195)**														
	Reward	—		—		—		.301 (.183)			.151 to .879		.006	
	Community	.246 (.181)		.049 to .769		.03		—			—		—	
	Value	.199 (.155)		–.031 to .586		.08		—			—		—	
	Risk perception	.269 (.175)		.144 to .837		.006		—			—		—	
	Worklife	—		—		—		.205 (.154)			–.011 to .601		.06	

^a^Only the factors remaining in the final backward elimination model are shown in the table.

^b^RN: registered nurse.

^c^PCP: primary care provider.

^d^Not applicable.

## Discussion

### Principal Findings

This study identified several aspects of the work environment associated with burnout for Korean American RNs and PCPs. As mentioned previously, the Job Demands-Resources Theory states that job demands such as heavy workload contribute to HCP burnout, whereas job resources, such as resource availability and social support, as well as recognition (reward) from peers and supervisors, contribute to professional well-being [22-24], which our findings support.

### Korean American RNs

Higher workload was associated with higher emotional exhaustion and depersonalization for RNs. With higher workloads come increased nurse encounters with patients and their caregivers and families, which may potentiate higher levels of emotional exhaustion and depersonalization. This may be particularly related to “emotional dirty work,” including diffusing charged patient interactions and other nuanced components of nursing tasks [35-37].

Emotionally draining or contentious interactions with patients’ families changed for nursing staff in the era of COVID-19. Although many hospitals sought to limit the number of visitors, nurses had to find creative ways to support patient-family interactions through phone- or web-based platforms [38]. Additionally, increased physical barriers were created by personal protective equipment (PPE), which limited the visibility of nurses’ facial expressions and discouraged other means of nonverbal shows of support, such as casual touching [39,40], thus creating additional communication barriers.

Furthermore, many nurses themselves were dealing with increased patient assignments from their coworkers calling in sick or from dealing with childcare or other issues resulting from the pandemic. This may possibly provide additional context for how our participants interpreted the concept of “workload.”

Higher risk perception and lower resource availability were also associated with higher emotional exhaustion, which has also been shown in other studies [41,42]. The critical shortage of N95 masks and other PPEs at the beginning of the pandemic, as well as risk perception including concerns of transmitting COVID-19 to their families or communities, may have contributed to the higher RN emotional exhaustion.

Our findings also show that a greater sense of community is associated with higher personal accomplishment for Korean American RNs, which may help mitigate the negative effects of burnout. In a highly hierarchy-oriented culture, wherein someone’s role in the organization determines their “status,” established rules (often related to age) may prevent younger (or novice) employees from approaching older, high-status employees for guidance and support, and younger employees may be more susceptible to workplace bullying [43]. Despite some modern shifts, perceived or actual traditional hierarchies within Korean culture often pervade the workplace [43]. Particularly among Korean American nurses in our study, the appreciation for nursing communities may be due to the ability of these groups to challenge hierarchy by creating safe spaces for nurses of all backgrounds and experience levels.

Another interesting finding from this study was the association between higher risk perception and higher personal accomplishment. The association between higher risk perception and higher emotion exhaustion is often a common finding across studies [41,42]; however, the link between higher risk perception and higher personal accomplishment is unexpected. A potential explanation may be that despite the high risks, nurses’ ability to commit to patients on the front lines during the COVID-19 pandemic may have instilled a higher sense of personal accomplishment for the participants of this study.

### Korean American PCPs

For PCPS, higher workload and poor sense of worklife were associated with higher emotional exhaustion and depersonalization, whereas higher reward or recognition was associated with personal accomplishment. PCPs may feel particularly vulnerable to higher emotional exhaustion and depersonalization, reflecting a high volume of patient encounters associated with the heightened need for and concurrent deficit of independent practitioners during the COVID-19 pandemic [44]. For providers and ancillary staff, it is also possible that the effects of the “great resignation,” a social phenomenon wherein a large number of employees left their job in 2021, may have exacerbated certain negative aspects of insufficient nonclinical support staff. Specifically, about 25% of Asian adults in a study conducted by the Pew Research Center reported quitting a job in 2021, compared with 17% of White adults, which may possibly have impacted the clinical environment in which these Korean American PCPs practiced [45].

Reward, which is also conceptualized as financial and social recognition for contributions on the job [33], was associated with higher personal accomplishment for the Korean American PCPs in this study. A sense of personal accomplishment may be achieved through a validation of one’s work or effort from their colleagues or certifications, awards, presentation opportunities, and promotions within the health care organization or professional organizations.

Similar to nurses, physicians have been recognized and hailed as health care “heroes” throughout the pandemic for their selfless acts and bravery. Although these PCPs certainly deserve much praise and attention, they deserve recognition in a way that is meaningful and enhances a sense of value to them. For instance, the “7 PM applause for health care heroes” initiative across the nation was a good way to show appreciation initially; however, it is not a sustainable strategy, nor does it address the underlying issue of HCP burnout. Furthermore, the term “health care heroes” masks the normalization of PCPs’ risk of exposure to the virus [46] and other ongoing emotional, psychological, and ethical issues associated with both the COVID-19 and burnout pandemics. An ongoing organization culture of recognition comprised of both personal praise with formal recognition and including multiple platforms for giving and receiving recognition may be the optimal solution for all HCPs.

### Implications

#### Professional Organizations

Nursing organizations such as the Korean American Nurses Association of Southern California, Asian American Pacific Islander Nurse Association, and National Coalition of Ethnic Minority Nurse Associations may serve as a professional community to share knowledge and resources; reward, recognize, and celebrate accomplishments; and help to minimize structural, societal, and cultural barriers contributing to burnout. Education and training opportunities may include assertiveness or generational differences in the workplace training, professional or leadership development including navigating the organizational hierarchy, effective communication, and the importance of self-care. In addition, they may also promote nurturing mentoring relationships as well as fellowship and networking opportunities including in-person (while observing COVID-19 precautions as needed) or web-based social hours, hiking or nature walks, and peer support groups for Korean American nurses by Korean American nurses.

Likewise, organizations such as the Korean American Medical Association of Southern California and Korean American Graduate Medical Association may serve as a professional community to help promote similar opportunities for Korean American PCPs. For instance, in a report by the American Medical Association on the experiences of ethnic minority physicians in the United States during the COVID-19 pandemic, Asian physicians reported that access to support and fellowship with others of similar demographic backgrounds would improve the sustainment of their well-being [47]. Additionally, 44.8% of Asian physicians reported that advocacy opportunities to address health inequities, particularly related to the COVID-19 pandemic, would also help their ability to sustain their well-being [47]. Furthermore, being a member of a professional organization can also serve as a form of social proof or recognition of one’s expertise and credibility in their field. It can also provide access to resources, training, and professional development opportunities as well as provide opportunities to recognize and celebrate personal accomplishments, which may be an effective burnout mitigation strategy, particularly for this group of Korean American PCP participants.

#### Community Partners

Moreover, these professional organizations may collaborate with community partners to provide comprehensive HCP wellness programs (eg, mindfulness, meditation, yoga, mental health counseling, stress or mental health first aid, and other activities) to help mitigate the adverse effects of burnout for Korean American HCPs. For instance, Korean American professional nursing and medical associations may collaborate with Korean community organizations such as the Korean Cultural Center, Los Angeles, to implement programs (eg, calligraphy and martial arts) for Korean American HCPs to promote overall well-being.

Furthermore, a community website or repository where Korean American HCPs and professional organizations can collaborate with community partners to share timely and accurate health information would benefit both HCPs and the community alike. According to a recent systemic review, up to 28.8% of social media posts about COVID-19 could be classified as misinformation [48]. Such incorrect information or misinformation not only jeopardizes measures to control the pandemic, but it also diverts resources and actions away from much needed communities. A community-based repository with translated materials (ie, in Korean) for HCPs and patients as well as a central location for shared resources (eg, masks and hand sanitizer donations) would be an effective strategy that enhances resource availability, builds a strong sense of community, and promotes well-being for both Korean American HCPs and the community.

#### Health Care Organization Partners

Targeted strategies for nurses and PCPs at the health care systems level, such as the distribution of fair workload and opportunities for all members of the health care team to practice at the top of their scope and being involved in decision-making opportunities, may improve a sense of control over their practice. Shared leadership councils, flexible work schedules, various nursing models, and the use of technology-based workers or assistants have been explored as potential strategies to promote nursing participation in decision-making processes, improve nursing workflow, and reduce workload.

Although PPE and other resource shortages are currently not as critical as they were at the start of the COVID-19 pandemic, health care organizations must remain diligent and be prepared for whatever future catastrophes that they may face. Regular inventory checks of essential health care resources both within the health care organization and throughout the community are critical. Additionally, routine incident command system and emergency management training in collaboration with local ethnic communities may be helpful, especially in meeting the needs of culturally and linguistically diverse populations.

#### Research

There are several implications for future research. First, just as there is a critical need for culturally congruent care provided by HCPs with similar cultural backgrounds to the patients they care for, there is a need for more culturally congruent research to be conducted by ethnic minority researchers who share the same cultural background as their participants. Such researchers are more likely to understand the cultural context and nuance of the research topic. This can be particularly important when researching culturally sensitive topics, for instance, when the research involves Korean cultural practices or beliefs or making practical and impactful recommendations specific to Korean American HCPs.

Along these lines, research focusing on ethnic minority groups should closely examine potential factors that impact health disparities and parse out the variations and nuances that exist within these groups. For instance, lumping all Asian American groups together—that is, combining the Korean American group with others from East, South, and Central Asia—may not only miss important subcultural differences but also mask meaningful differences in health risks among these groups, including mental health–related risk factors. Therefore, future studies on ethnic minority groups should consider the cultural and sociodemographic heterogeneity as well as variations in health risk factors that distinguish each subgroup before aggregating them into one group.

Finally, more funding opportunities aimed at exploring identity-informed burnout experiences of HCPs and studies aimed at capturing nuances both across and within ethnic minority groups are needed. The COVID-19 pandemic has exasperated already high levels of burnout among HCPs around the globe. Supporting HCP well-being requires continuous investment in burnout research and information sharing to advance evidence-based solutions [49], contextualized within the HCPs’ cultural, racial/ethnic, and other socially informed or self-selected group memberships.

### Limitations

The study participants were recruited among Korean American nurses and PCPs practicing in Southern California; therefore, the findings from this study may not be generalizable to all nurses and PCPs. Additionally, the total study population could not be confirmed, especially as the participants were referred to the study via word of mouth from other participants. Consequently, the response rates could not be confirmed. Nonetheless, the methods applied and lessons learned from this study may guide further studies that apply more rigorous research methods to evaluate the work environment’s impact on burnout and the well-being of nurses and PCPs from various ethnic minority groups.

Furthermore, the assessment of Korean American nurses’ and PCPs’ experience with discrimination, such as “Asian Hate,” during the pandemic and its impact on burnout was not within the scope of this study. Although we draw attention to the potential relationship between these factors among our study participants, further studies specifically assessing their experience with discrimination are needed to make direct correlations between nurse and PCP discrimination and burnout. Additional aspects of personal identity beyond ethnic/racial group, such as generation in the United States, language preference, and level of acculturation, among others, would allow for a more refined view of this experience among Korean American nurses and PCPs and should be considered in future iterations of this work.

### Conclusions

Three years into the start of the pandemic, the COVID-19 endemic seems more hopeful than the burnout endemic. Findings from this study underscore the importance of a multilevel nurse and PCP wellness program sponsored by professional organizations, communities, and the health care institutions in which these nurses and PCPs practice. Finally, a growing recognition of identity-informed burnout experiences across frontline Korean American nurses and PCPs argues for future explorations that capture nuance both across and within this and other ethnic minority nurse and PCP groups. By recognizing and capturing these variations, we may better support the creation of targeted, burnout-mitigating strategies for all.

## Appendix

Multimedia Appendix 1 Bivariate Pearson correlation analysis.

## Abbreviations

AWS: Areas of Worklife Survey
HCP: health care provider
MBI-HSS: Maslach Burnout Inventory–Human Services Survey
MD: Doctor of Medicine
NP: nurse practitioner
PA: physician assistant
PEPS: Pandemic Experience & Perceptions Survey
PPE: Personal protective equipment
REDCap: Research Electronic Data Capture
RN: registered nurse
