# Supplementary material for: The Association Between Korean American Nurse and Primary Care Provider Burnout, Areas of Worklife, and Perceptions of Pandemic Experience: Cross-sectional Study
Source: Asian Pac Isl Nurs J. 2023 Mar 8;7:e42490. doi: 10.2196/42490 (PMC10034608; doi:10.2196/42490)
Supplement: Multimedia Appendix 1 [file apinj_v7i1e42490_app1.docx]

**Multimedia Appendix 1**

Bivariate Pearson Correlation Analysis: RN vs. PCP: Factors Associated with Burnout (Emotional Exhaustion, Depersonalization, and Personal Accomplishment)

| **RN Variable** | | | | Emotion-al Ex-  haustion | | De-person-alization | Personal Accomp-lishment | Work-Load | Control | Re-ward | Comm-unity | Fair-  ness | Value | Re-sources | Risk Per-ception | Work-Life | Leader-  ship |
| --- | --- | --- | --- | --- | --- | --- | --- | --- | --- | --- | --- | --- | --- | --- | --- | --- | --- |
| **Emotional exhaustion** | | | | | | | | | | | | | | | | | |
|  | | *r* | | 1 | | .645^c^ | .055 | .492^c^ | -.096 | -.364^c^ | -.175 | -.315^b^ | -.196 | -.347^c^ | .321^b^ | -.167 | -.211^a^ |
|  |  | *P* value | | -^d^ | | <.001 | .59 | <.001 | .35 | <.001 | .09 | .002 | .05 | <.001 | .001 | .10 | .04 |
| **Depersonalization** | | | | | | | | | | | | | | | | | |
|  | | *r* | | .645^c^ | | 1 | -.041 | .302^b^ | .117 | -.198 | -.114 | -.142 | -.088 | -.159 | .194 | -.096 | -.079 |
|  |  | *P* value | | <.001 | | - | .69 | .003 | .25 | .05 | .264 | .17 | .39 | .12 | .06 | .35 | .44 |
| **Personal accomplishment** | | | | | | | | | | | | | | | | | |
|  | | *r* | | .055 | | -.041 | 1 | -.113 | .199 | .135 | .333^b^ | -.012 | .265^b^ | .066 | .211^a^ | .190 | .232^a^ |
|  |  | *P* value | | .59 | | .69 | - | .27 | .05 | .19 | .001 | .91 | .009 | .52 | .04 | .06 | .02 |
| **Workload** | | | | | | | | | | | | | | | | | |
|  | | *r* | | .492^c^ | | .302^b^ | -.113 | 1 | -.271^b^ | -.365^c^ | -.108 | -.411^c^ | -.343^b^ | -.354^c^ | .194 | -.225^a^ | -.235^a^ |
|  |  | *P* value | | <.001 | | .003 | .27 | - | .007 | <.001 | .29 | <.001 | .001 | <.001 | .06 | .03 | .02 |
| **Control** | | | | | | | | | | | | | | | | | |
|  | | *r* | | -.096 | | .117 | .199 | -.271^b^ | 1 | .402^c^ | .472^c^ | .378^c^ | .517^c^ | .393^c^ | -.216^a^ | .491^c^ | .380^c^ |
|  |  | *P* value | | .35 | | .25 | .05 | .007 | - | <.001 | <.001 | <.001 | <.001 | <.001 | .03 | <.001 | <.001 |
| **Reward** | | | | | | | | | | | | | | | | | |
|  | | *r* | | -.364^c^ | | -.198 | .135 | -.365^c^ | .402^c^ | 1 | .461^c^ | .511^c^ | .412^c^ | .405^c^ | -.237^a^ | .477^c^ | .499^c^ |
|  |  | *P* value | | <.001 | | .05 | .19 | <.001 | <.001 | - | <.001 | <.001 | <.001 | <.001 | .02 | <.001 | <.001 |
| **Community** | | | | | | | | | | | | | | | | | |
|  | | *r* | | -.175 | | -.114 | .333^b^ | -.108 | .472^c^ | .461^c^ | 1 | .461^c^ | .513^c^ | .384^c^ | -.054 | .501^c^ | .506^c^ |
|  |  | *P* value | | .09 | | .26 | .001 | .29 | <.001 | <.001 | - | <.001 | <.001 | <.001 | .60 | <.001 | <.001 |
| **Fairness** | | | | | | | | | | | | | | | | | |
|  | | *r* | | -.315^b^ | | -.142 | -.012 | -.411^c^ | .378^c^ | .511^c^ | .461^c^ | 1 | .624^c^ | .618^c^ | -.199 | .573^c^ | .569^c^ |
|  |  | *P* value | | .002 | | .17 | .91 | <.001 | <.001 | <.001 | <.001 | - | <.001 | <.001 | .05 | <.001 | <.001 |
| **Value** | | | | | | | | | | | | | | | | | |
|  | | *r* | | -.196 | | -.088 | .265^b^ | -.343^b^ | .517^c^ | .412^c^ | .513^c^ | .624^c^ | 1 | .511^c^ | -.224^a^ | .579^c^ | .532^c^ |
|  |  | *P* value | | .05 | | .39 | .009 | .001 | <.001 | <.001 | <.001 | <.001 | - | <.001 | .027 | <.001 | <.001 |
| **Resource** | | | | | | | | | | | | | | | | | |
|  | | *r* | | -.347^c^ | | -.159 | .066 | -.354^c^ | .393^c^ | .405^c^ | .384^c^ | .618^c^ | .511^c^ | 1 | -.220^a^ | .635^c^ | .625^c^ |
|  |  | *P* value | | <.001 | | .12 | .52 | <.001 | <.001 | <.001 | <.001 | <.001 | <.001 | - | .030 | <.001 | <.001 |
| **Risk Perception** | | | | | | | | | | | | | | | | | |
|  | | *r* | | .321^b^ | | .194 | .211^a^ | .194 | -.216^a^ | -.237^a^ | -.054 | -.199 | -.224^a^ | -.220^a^ | 1 | -.288^b^ | -.173 |
|  |  | *P* value | | .001 | | .06 | .04 | .06 | .03 | .02 | .60 | .05 | .03 | .030 | - | .004 | .09 |
| **Worklife** | | | | | | | | | | | | | | | | | |
|  | | *r* | | -.167 | | -.096 | .190 | -.225^a^ | .491^c^ | .477^c^ | .501^c^ | .573^c^ | .579^c^ | .635^c^ | -.288^b^ | 1 | .701^c^ |
|  |  | *P* value | | .10 | | .35 | .06 | .03 | <.001 | <.001 | <.001 | <.001 | <.001 | <.001 | .004 | - | <.001 |
| **Leadership** | | | | | | | | | | | | | | | | | |
|  | | *r* | | -.211^a^ | | -.079 | .232^a^ | -.235^a^ | .380^c^ | .499^c^ | .506^c^ | .569^c^ | .532^c^ | .625^c^ | -.173 | .701^c^ | 1 |
|  |  | *P* value | | .04 | | .44 | .02 | .02 | <.001 | <.001 | <.001 | <.001 | <.001 | <.001 | .09 | <.001 | - |
| **PCP Variable** | | | Emotion-al Ex-  haustion | | De-person-alization | | Personal Accomp-lishment | Work-Load | Control | Re-ward | Comm-unity | Fair-  ness | Value | Re-sources | Risk Per-ception | Work-Life | Leader-  ship |
| **Emotional exhaustion** | | | | | | | | | | | | | | | | | |
|  | *r* | | 1 | | | .652^c^ | -.115 | .458^c^ | -.409^c^ | -.259^a^ | -.364^c^ | -.281^b^ | -.369^c^ | -.254^a^ | .094 | -.424^c^ | -.290^b^ |
|  | *P* value | | - ^d^ | | | <.000 | .29 | <.000 | <.000 | .02 | <.000 | .008 | <.000 | .02 | .39 | <.000 | .006 |
| **Depersonalization** | | | | | | | | | | | | | | | | | |
|  | *r* | | .652^c^ | | | 1 | -.261^a^ | .376^c^ | -.230^a^ | -.249^a^ | -.205 | -.085 | -.367^c^ | -.270^a^ | .146 | -.454^c^ | -.279^b^ |
|  | *P* value | | <.000 | | | - | .01 | <.000 | .03 | .02 | .05 | .43 | <.000 | .01 | .18 | <.000 | .009 |
| **Personal accomplishment** | | | | | | | | | | | | | | | | | |
|  | *r* | | -.115 | | | -.261^a^ | 1 | -.231^a^ | .324^b^ | .388^c^ | .334^b^ | .240^a^ | .204 | .118 | -.026 | .321^b^ | .164 |
|  | *P* value | | .29 | | | .01 | - | .03 | .002 | <.000 | .001 | .03 | .06 | .28 | .81 | .002 | .13 |
| **Workload** | | | | | | | | | | | | | | | | | |
|  | *r* | | .458^c^ | | | .376^c^ | -.231^a^ | 1 | -.364^b^ | -.177 | -.094 | -.056 | -.275^a^ | -.294^b^ | .059 | -.313^**^ | -.365^b^ |
|  | *P* value | | <.000 | | | <.000 | .03 | - | .001 | .10 | .386 | .61 | .01 | .006 | .58 | .003 | .001 |
| **Control** | | | | | | | | | | | | | | | | | |
|  | *r* | | -.409^*c^ | | | -.230^a^ | .324^b^ | -.364^b^ | 1 | .438^c^ | .479^c^ | .485^c^ | .471^c^ | .153 | -.010 | .539^c^ | .417^c^ |
|  | *P* value | | . <.000 | | | .03 | .002 | .001 | - | <.000 | <.000 | <.000 | <.000 | .16 | .93 | <.000 | <.000 |
| **Reward** | | | | | | | | | | | | | | | | | |
|  | *r* | | -.259^a^ | | | -.249^a^ | .388^c^ | -.177 | .438^c^ | 1 | .536^c^ | .448^c^ | .368^c^ | .197 | -.010 | .386^c^ | .419^c^ |
|  | *P* value | | .02 | | | .02 | <.000 | .100 | <.000 | - | <.000 | <.000 | <.000 | .07 | .92 | <.000 | <.000 |
| **Community** | | | | | | | | | | | | | | | | | |
|  | *r* | | -.364^c^ | | | -.205 | .334^b^ | -.094 | .479^c^ | .536^c^ | 1 | .663^c^ | .592^c^ | .328^b^ | -.143 | .535^c^ | .538^c^ |
|  | *P* value | | <.000 | | | .06 | .001 | .39 | <.000 | <.000 | - | <.000 | <.000 | .002 | .19 | <.000 | <.000 |
| **Fairness** | | | | | | | | | | | | | | | | | |
|  | *r* | | -.281^b^ | | | -.085 | .240^a^ | -.056 | .485^c^ | .448^c^ | .663^c^ | 1 | .669^c^ | .430^c^ | -.102 | .577^c^ | .541^c^ |
|  | *P* value | | .008 | | | .43 | .03 | .61 | <.000 | <.000 | <.000 | - | <.000 | <.000 | .35 | <.000 | <.000 |
| **Value** | | | | | | | | | | | | | | | | | |
|  | *r* | | -.369^c^ | | | -.367^c^ | .204 | -.275^a^ | .471^c^ | .368^c^ | .592^c^ | .669^c^ | 1 | .407^c^ | -.101 | .715^c^ | .705^c^ |
|  | *P* value | | <.000 | | | <.000 | .06 | .01 | <.000 | <.000 | <.000 | <.000 | - | <.000 | .35 | <.000 | <.000 |
| **Resource** | | | | | | | | | | | | | | | | | |
|  | *r* | | -.254^a^ | | | -.270^a^ | .118 | -.294^b^ | .153 | .197 | .328^b^ | .430^c^ | .407^c^ | 1 | -.161 | .474^**^ | .467^c^ |
|  | *P* value | | .02 | | | .01 | .28 | .006 | .16 | .07 | .002 | <.000 | <.000 | - | .14 | <.000 | <.000 |
| **Risk Perception** | | | | | | | | | | | | | | | | | |
|  | *r* | | .094 | | | .146 | -.026 | .059 | -.010 | -.010 | -.143 | -.102 | -.101 | -.161 | 1 | -.199 | -.198 |
|  | *P* value | | .39 | | | .18 | .81 | .58 | .93 | .92 | .19 | .35 | .35 | .14 | - | .06 | .07 |
| **Worklife** | | | | | | | | | | | | | | | | | |
|  | *r* | | -.424^c^ | | | -.454^c^ | .321^b^ | -.313^b^ | .539^c^ | .386^c^ | .535^c^ | .577^c^ | .715^*c^ | .474^c^ | -.199 | 1 | .648^c^ |
|  | *P* value | | <.000 | | | <.000 | .002 | .003 | <.000 | <.000 | <.000 | <.000 | <.000 | <.000 | .064 | - | <.000 |
| **Leadership** | | | | | | | | | | | | | | | | | |
|  | *r* | | -.290^b^ | | | -.279^b^ | .164 | -.365^b^ | .417^c^ | .419^c^ | .538^c^ | .541^c^ | .705^c^ | .467^c^ | -.198 | .648^c^ | 1 |
|  | *P* value | | .006 | | | .009 | .13 | .001 | <.000 | <.000 | <.000 | <.000 | <.000 | <.000 | .07 | <.000 | - |

a. The correlation is significant at a significance level of <.05 (two-tailed).

b. The correlation is significant at a significance level of <.01 (two-tailed).

c. The correlation is significant at a significance level of <.001 (two-tailed).

d. Not applicable.
